# Novel Biomaterials Used in Medical 3D Printing Techniques

**DOI:** 10.3390/jfb9010017

**Published:** 2018-02-07

**Authors:** Karthik Tappa, Udayabhanu Jammalamadaka

**Affiliations:** Mallinckrodt Institute of Radiology, Washington University School of Medicine, Saint Louis, MO 63110, USA; udayabhanuj@gmail.com

**Keywords:** additive manufacturing, 3D printing, biomaterials, customized implants, tissue engineering, regenerative medicine, drug delivery, fused deposition modeling, bioprinting, inkjet, polyjet

## Abstract

The success of an implant depends on the type of biomaterial used for its fabrication. An ideal implant material should be biocompatible, inert, mechanically durable, and easily moldable. The ability to build patient specific implants incorporated with bioactive drugs, cells, and proteins has made 3D printing technology revolutionary in medical and pharmaceutical fields. A vast variety of biomaterials are currently being used in medical 3D printing, including metals, ceramics, polymers, and composites. With continuous research and progress in biomaterials used in 3D printing, there has been a rapid growth in applications of 3D printing in manufacturing customized implants, prostheses, drug delivery devices, and 3D scaffolds for tissue engineering and regenerative medicine. The current review focuses on the novel biomaterials used in variety of 3D printing technologies for clinical applications. Most common types of medical 3D printing technologies, including fused deposition modeling, extrusion based bioprinting, inkjet, and polyjet printing techniques, their clinical applications, different types of biomaterials currently used by researchers, and key limitations are discussed in detail.

## 1. Introduction

**Figure 1:**
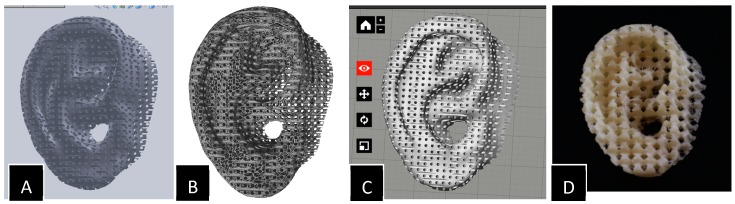
Sequential steps involved in a 3D printing process. (**A**) Designed 3D computer aided design (CAD) model; (**B**) Stereolithography (STL) file of the model; (**C**) Slicing or 3D printing software; (**D**) 3D printed object.

Three-dimensional printing is a process of building 3D objects from a digital file. In this process, a digital 3D object is designed using computer aided design (CAD) software. SolidWorks, AutoCAD, and ZBrush are some examples of popular CAD software used commercially in industries. Blender, FreeCAD, Meshmixer, and SketchUp are some examples of the freeware commonly used to make 3D models. These 3D objects are saved in a 3D printer-readable file format. The most common universal file formats used for 3D printing are STL (stereolithography) and VRML (virtual reality modeling language). Additive manufacturing file format (AMF), GCode, and ×3g are some of the other 3D printer readable file formats. Figure 1 shows the steps involved in 3D printing of an object from a CAD design.

In additive manufacturing, material is laid in layer-by-layer fashion in the required shape, until the object is formed. Although the term 3D printing is used as a synonym for additive manufacturing, there are several different fabricating processes involved in this technology. Depending on the 3D printing process, additive manufacturing can be classified into four categories, including extrusion printing, material sintering, material binding, and object lamination. Table 1 shows a broad classification of the different types of 3D printing techniques and their working principles.

The 3D printing technology has been in use more than three decades in the automobile and aeronautical industries. In the medical field, the use of this technology was limited only to 3D printing of anatomical models for educational training purposes. Only with the recent advancements in developing novel biodegradable materials has the use of 3D printing in medical and pharmaceutical fields boomed. Today, additive manufacturing technology has wide applications in the clinical field and is rapidly expanding. It has revolutionized the healthcare system by customizing implants and prostheses, building biomedical models and surgical aids personalized to the patient, and bioprinting tissues and living scaffolds for regenerative medicine. Table 2 shows the applications of 3D printing technology in various sectors.

Biomaterials are natural or synthetic substances that are in contact with biological systems, and help to repair, replace, or augment any tissue or organ of the body for any period of time. Based on the chemical nature of the substances, biomaterials used in 3D printing are broadly classified into four categories, as show in Table 3. An ideal 3D printing biomaterial should be biocompatible, easily printable with tunable degradation rates, and morphologically mimic living tissue.

The selection of biomaterial for a 3D printing mechanism depends on the application of end product. For instance, biomaterial used for orthopedic or dental applications should have high mechanical stiffness and prolonged biodegradation rates. By contrast, for dermal or other visceral organ applications, the biomaterial used should be flexible and have faster degradation rates. The majority of biomaterials used in current medical 3D printing technology, such as metals, ceramics, hard polymers, and composites, are stiff, and thus widely used for orthodontic applications. Soft polymers, including hydrogels, are widely used in bioprinting cells for tissue/organ fabrication. The hydrogel microenvironment mimics the extracellular matrix of a living tissue, and thus, cells are easily accommodated.

## 2. Commonly Used 3D Printing Technologies in the Medical Field

Among the various types of 3D printing techniques described in the Table 1, FDM, extrusion based bioprinting, inkjet, and polyjet are the most common types of additive manufacturing techniques used in the medical field.

### 2.1. Fused Deposition Modeling (FDM) or Free Form Fabriction (FFF)

**Figure 2:**
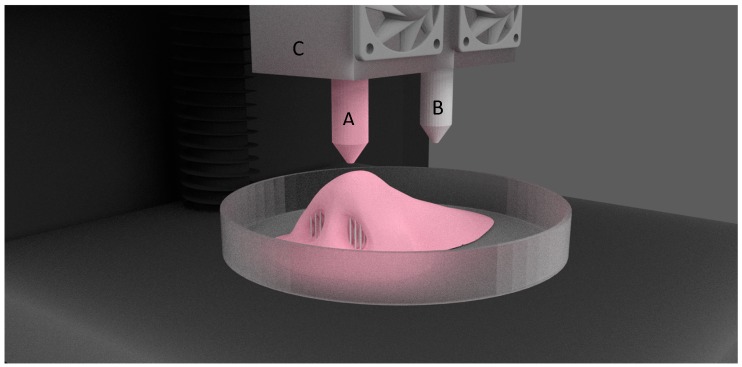
Dual head FDM 3D printer. (**A**) Building material; (**B**) Supporting material; (**C**) Print heads.

FDM is the most common and inexpensive type of additive manufacturing technology. In this technique, a thermoplastic filament is passed through a heated print head and is laid down on to the build platform in layer-by-layer fashion, until the required object is formed. MakerBot, Ultimaker, Flashforge, and Prusa are some of the commercially available inexpensive desktop 3D printers. These printers are limited by the variety of the materials being used, and produce lower resolution objects. Expensive FDM printers, which can use wide varieties of materials and can print at higher resolutions are also available, such as Stratasys 3D printers. FDM printers can accommodate more than one print head, and thus, can print multiple types of materials at a time. Usually, among these multi-head printers, one of the print head bears a supporting filament which can be easily removed or dissolved in water. Figure 2 shows the parts of FDM 3D printer.

ABS is the most common thermoplastic polymer used for FDM process. PLA, nylon, polycarbonate (PC), and polyvinyl alcohol (PVA) are some of the other commonly used printing filaments. Lactic acid-based polymers, including PLA and PCL, are well known for their biocompatible and biodegradable properties, and hence, are extensively used for medical and pharmaceutical applications. Additionally, PLA and PCL melt at low temperatures, 175 °C and 65 °C respectively, making it easy to load drugs without losing their bioactivity due to thermal degradation. These polymers undergo hydrolysis in vivo, and are eliminated through excretory pathways [6,7]. Comparatively, PCL has lower mechanical strength than PLA, and thus, used for non-load bearing applications.

Printing parameters, such as raster angle, raster thickness, and layer height, play a crucial role in fabricating biocompatible scaffolds with required pore size and mechanical strength. Combinations of materials, such as PCL/chitosan [8] or PCL/β-TCP (tricalcium phosphate) [9] are also used in the FDM process to enhance the bioactive properties of the scaffolds. FDM has the ability to build constructs quickly, with dimensional accuracy and excellent mechanical properties. Hence it is used widely for prototyping in industry. In medicine, FDM is used for fabricating customized patient-specific medical devices, such as implants, prostheses, anatomical models, and surgical guides. Various thermoplastic polymers are doped with variety of bioactive agents, including antibiotics [10], chemotherapeutics [11], hormones [12], nanoparticles [13,14], and other oral dosages [15,16] for personalized medicine. Using this technology, non-biocompatible materials, such as ABS [17] or thermoplastic polyurethane (TPU), are used for creating medical models for perioperative surgical planning and simulations [18]. These models are also used as a tool to explain the procedures to the patients before they undergo surgery. Table 4 shows the types of biomaterials used in FDM technique for clinical applications.

### 2.2. Extrusion Based Bioprinting

**Figure 3:**
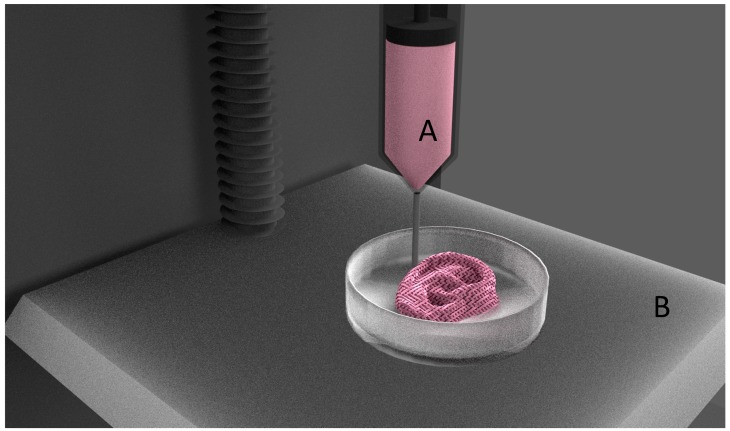
Extrusion based bioprinting. (**A**) Bioink; (**B**) Build platform.

In this method, materials are extruded through a print head either by pneumatic pressure or mechanical force. Similar to FDM, materials are continuously laid in layer-by-layer fashion until the required shape is formed, as shown in Figure 3. Since this process does not involve any heating procedures, it is most commonly used for fabricating tissue engineering constructs with cells and growth hormones laden. Bioinks are the biomaterials laden with cells and other biological materials, and used for 3D printing. This 3D printing process allows for the deposition of small units of cells accurately, with minimal process-induced cell damage. Advantages such as precise deposition of cells, control over the rate of cell distribution and process speed have greatly increased the applications of this technology in fabricating living scaffolds.

A wide range of materials with varied viscosities and high cell density aggregates can be 3D printed using this technique. A large variety of polymers are under research for the use in bioprinting technology. Natural polymers, including collagen [20], gelatin [21], alginate [22], and hyaluronic acid (HA) [23], and synthetic polymers, such as PVA [24] and polyethylene glycol (PEG), are commonly used in bioinks for 3D printing. Often these bioinks are post-processed either by chemical or UV crosslinking to enhance the constructs mechanical properties. Depending on the type of polymer used in the bioink, biological tissues and scaffolds of varied complexity can be fabricated. Multiple print heads carrying different types of cell lines for printing a complex multicellular construct can be possible with this technique. Lee et al., have used six extrusion headed 3D printer with six different bioinks, including PEG as a sacrificial ink to fabricate a living human ear [25]. Laronda et al., has used this extrusion bioprinting to fabricate gelatin based ovarian implants which can accommodate ovarian follicles. These implants restored the ovarian functions of the sterilized mice, and they even bore offspring [21].

Extrusion bioprinting has been used for fabricating scaffolds for regeneration of bone [26], cartilage [22], aortic valve [27], skeletal muscle [28], neuronal [29], and other tissues. In spite of all this success, material selection and mechanical strength still remains a major concern for bioprinting. Fabricating vascularization within a complex tissue is still an unanswered problem faced by this technology. To address this issue, researchers have focused on using sacrificial materials, which are incorporated within the construct while 3D printing, and are removed in post-processing, leaving the void spaces to act as vascularization channels [30]. Table 5 shows some of the biomaterials currently used by researchers, and their applications.

### 2.3. Material Sintering

In material sintering type of 3D printing technique, the powdered form of printing material in a reservoir is fused into a solid object, either by using physical (UV/laser/electron beam) or chemical (binding liquid) sources. SLA type is the oldest and widely used technology among metal sintering 3D printers. Unlike extrusion based printers, there is no contact between the print head and printing object. The objects can be 3D printed with high accuracy and resolution with this technique. The major limitation of this technology includes limited availability of photocurable polymer resins. Majority of the SLA resins currently available are based on low molecular weight polyacrylate or epoxy resins. For biomedical applications, polymer ceramic composite resins, made up of hydroxyapatite based calcium phosphate salts, are commonly used.

### 2.4. Inkjet or Binder Jet Printing

**Figure 4:**
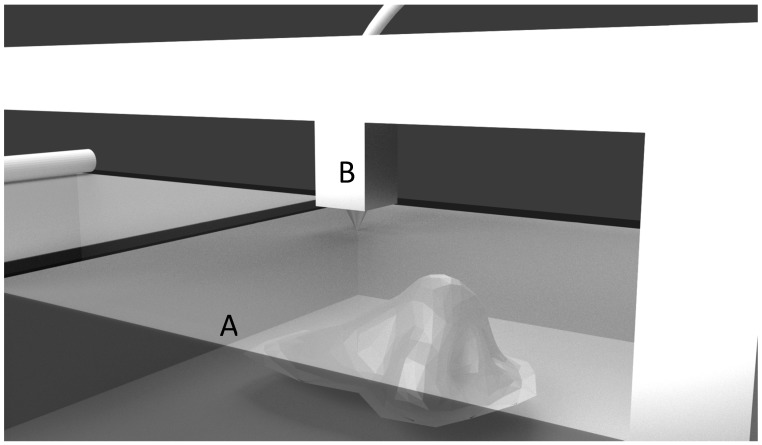
Inkjet 3D printing. (**A**) Powdered bed; (**B**) Binding liquid spraying nozzle.

This process is similar to SLS; instead of fusing the powder bed with laser or electron beam, binding liquid is selectively dropped on to the powdered bed to bind the materials in a layer-by-layer fashion as shown in Figure 4. This process is continued until the final object is formed. Thermal and piezoelectric are two types of printing heads used in this technique. In thermal print head systems, an electric heating unit is present inside the deposition head, which vaporizes the binding material to form a vapor bubble. This vapor bubble expands due to pressure, and comes out of the print head as a droplet. Whereas in the piezoelectric print head system, the voltage pulse in the print head induces a volumetric change (changes in pressure and velocity) in the binder liquid, resulting in the formation of a droplet. These printers are known for their precise deposition of the binder liquid with speed and accuracy.

Water, phosphoric acid, citric acid, PVA, poly-DL-lactide (PDLLA) are some of the commonly used binding materials for inkjet 3D printing. A wide range of powdered substances, including polymers and composites, are used for medical and tissue engineering applications. Finished 3D printed objects are often post-processed to enhance the mechanical properties. Wang et al., have used phosphoric acid and PVA as binding liquids to bind HA/β-TCP powders for bone tissue regeneration applications. The accuracy and mechanical strength of constructs printed using phosphoric acid were higher than constructs printed using PVA [35]. Sandler et al., have fabricated precise and personalized dosage forms using concentrated solutions of paracetamol, theophylline, and caffeine [36]. Uddin et al., have surface coated metallic transdermal needles with chemotherapeutic agents using Soluplus, a copolymer of PVC–PVA–PEG, for transdermal drug delivery [37]. Table 6 shows the types of binding liquids and respective powder materials used for inkjet printing.

### 2.5. Polyjet Printing

**Figure 5:**
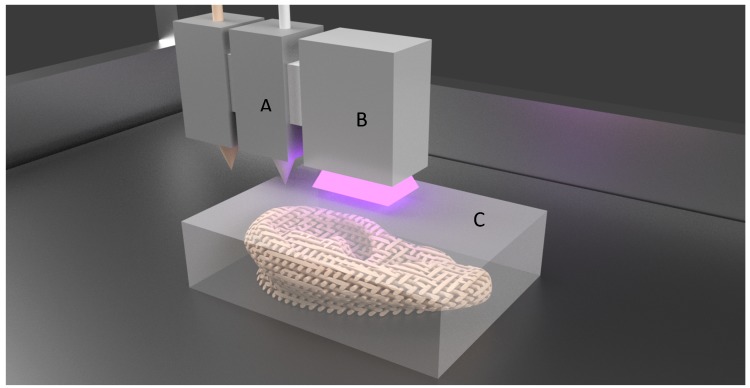
Polyjet 3D printer. (**A**) Nozzle spraying photopolymer; (**B**) UV source; (**C**) Supporting material.

Similar to inkjet printing, layers of photopolymer resin are jetted on to the build platform and are simultaneously cured using UV light source, as shown in Figure 5. Unlike inkjet process, multiple types of materials can be jetted simultaneously and cured. This gives us the ability to fabricate a complex multi-material object. Due to these capabilities, polyjet is widely used in the medical field to fabricate anatomical models for surgical planning and pre-operative simulations. High resolution objects with varied modular strengths can be 3D printed with high dimensional accuracy using polyjet technique. Since the UV source is right next to the jetting nozzle and cures the resin instantaneously, post-processing of the construct will not be necessitated. This technology is relatively new to the additive manufacturing field. Many types of photopolymers, such as ABS like, Veroclear, Verodent, and Fullcure are commercially available for use in polyjet printing. Table 7 shows some of the photopolymers used in medical applications.

### 2.6. Laminated Object Manufacturing

In this type of 3D printing technology, thin layers of paper, plastic, or metal sheets are glued together in layer-by-layer fashion, and cut into the required shape using a metallic cutter or laser. This process is inexpensive, fast, and easy to use. It fabricates relatively lower resolution objects and is used for multicolor prototyping.

## 3. Limitations

Although 3D printing has the ability to fabricate on-demand, highly personalized complex designs at low costs, this technology’s medical applications are limited due to lack of diversity in biomaterials. Even with the availability of variety of biomaterials including metals, ceramics, polymers, and composites, medical 3D printing is still confined by factors such as biomaterial printability, suitable mechanical strength, biodegradation, and biocompatible properties.

Usually, in extrusion based bioprinting, higher concentrations of polymers are used in fabricating bioinks to obtain structural integrity of the end product. This dense hydrogel environment limits the cellular network and functional integration of the scaffold. For any moderate sized biological scaffold to be functional, vascularization is of utmost importance, and is not possible with the current 3D printing technology. Small scale scaffolds currently printed in the laboratories of researchers can easily survive through diffusion, but a life-size functional organ must have a profuse vascularization. To address this problem, incorporation of sacrificial materials during the scaffold fabrication has been used by many researchers. These materials fill up the void spaces, providing mechanical support to the printing materials, and once constructs are fabricated, they are removed by post-processing methods. Many sacrificial/fugitive materials including carbohydrate glass [55], pluronic glass [56], and gelatin microparticles [57] are currently under investigation [5].

Additionally, design induced limitations cause material discontinuity, due to poor transformation of complex CAD design into machine instructions. Process induced limitations include differences in porosities of CAD object and finished 3D printed product [58].

## 4. Conclusions

In summary, 3D printing has been revolutionizing the medical field, and is still rapidly expanding. Popular clinical applications include fabrication of patient-specific implants and prostheses; engineering scaffolds for tissue regeneration and biosynthetic organs; personalization of drug delivery systems; and anatomical modeling for perioperative simulations. The use of 3D printing in the medical field is continuously growing, due to its capabilities, such as personalization of medicine, cost efficiency, speed, and enhanced productivity [59]. With the advancement in 3D modeling software and mechanics of the printing machine, the dimensional precision, speed, and tunability of a 3D printer has been vastly improved. Using finite element analysis, the change in the mechanical properties of the finished product with respect to printing parameters can be simulated, and best suiting parameters can be obtained beforehand. Even with all these advancements, medical 3D printing is still budding and has incredible potential.

Currently, there are only a limited number of biodegradable polymers available for 3D printing. Most of these 3D printing biomaterials are used for either drug delivery or space-filling implantation purposes. Therefore, there is a major need for research to fabricate novel biopolymers with tunable bio-properties and that can restore functionality at the site of application. Inexpensive, readily available lactic acid based polymers (such as PLA and PCL) are focused on, mainly due to their abilities to perform well in most types of 3D printing technologies. Additionally, they have excellent mechanical and biodegradable properties. These polymers are also mixed with traditional biomaterials (such as HA, TCP) and used as composites to provide higher printability, mechanical stability, and greater tissue integration for orthopedic applications.

With continuous research in bioprinting and biomaterials technology, we are getting closer to fabricating life-sized, fully functional 3D printed organs. Bioprinting is still in its early stages, where many researchers have proved the feasibility of 3D printing a functional organ in a laboratory. Soon, there will be an advancement in use of these biomaterials/bioinks from labs to clinical trials, and eventually, in everyday clinical practice. This could be a potential solution to address the problem of continuous organ donor’s shortage. Moreover, the ability of the 3D printer to fabricate tissues/organs from the host cells will reduce the immune response of the implant, and in turn, reduce tissue rejection.

## Figures and Tables

**Table 1:** Types of 3D printing technologies.

Process	Principle
Extrusion Printing	
Fused Deposition Modeling (FDM) [1]	A thermoplastic material is melted and laid on to the build platform in layer-by-layer fashion, until the object is formed.
	Materials: acrylonitrile butadiene styrene (ABS), poly-lactic acid (PLA), nylon.
Bioprinting [2]	Biological materials are extruded through a nozzle under pressure to lay down materials in sequential layers till the scaffold is built.
	Materials: alginate, chitosan, gelatin, collagen, fibrin.
Material Sintering	
Selective Laser Sintering (SLS) [3]	A high-power laser beam fuses the powdered materials in layer-by-layer pattern to form an object.
	Materials: nylon, polyamide.
Electron Beam Manufacturing (EBM)	EBM is similar to SLS, except for high power electron beam is used to fuse the powdered particles.
	Materials: titanium, cobalt−chrome alloy.
Stereolithography (SLA) [4]	A UV laser beam selectively hardens the photo-polymer resin in layers.
	Each layer is solidified and built on top of next until the object is formed.
	Materials: photopolymers.
Continuous Liquid Interface Production (CLIP) [3]	CLIP is similar to SLA, except for UV beam is passed through a transparent window at the bottom of the resin and build platform raises upwards holding the 3D printed object.
	Materials: photopolymers.
Material Binding	
Binder Jetting/Inkjet [5]	A liquid binding material is selectively dropped into the powder bed in alternative layers of powder–binding liquid–powder, until the final object is formed.
	Materials: starch or gypsum (powder bed) and water (binding agent)
Polyjet	Polyjet printing is similar to inkjet, but instead of binding agents, photopolymer liquid is sprayed in layers onto the build platform and is instantaneously cured using UV light.
	Materials: polypropylene, polystyrene, polycarbonate.
Lamination	
Laminated Object Manufacturing (LOM)	Layers of adhesive coated material are successively glued together and cut in required shapes using a laser.
	Materials: thin sheets of paper, polyvinyl caprolactam (PVC) plastic, or metal laminates

**Table 2:** Applications of 3D printing.

Sector	Applications
Industry	Jigs, fixtures, and end-use parts for aeronautical industry
	Prototypes and spare parts for automotive industry
Medical	Surgical models for perioperative surgical preparations
	Dental fixtures, bridges, and crowns
	Customized patient specific implants and prostheses
	Living tissue scaffolds for tissue engineering and regenerative medicine
Pharmaceutical	Customized implants for drug delivery
	Tablets, capsules, and other patient specific dosages
Food	Designing and 3D printing complex shaped cakes, cookies, candies, pizzas, and other desserts
Fashion	Jewelry, clothes, shoes, and other accessories
Household	Plates, cups, spoons, holders, and other common household objects
Miscellaneous	Space: building prototypes and parts in space
	Chemical industry: fabricating complex molecules and compounds
	Construction: scale models with intricate architectures

**Table 3:** Biomaterials classification with their advantages, disadvantages, and applications.

Type	Advantages	Disadvantages	Applications
Metals and metal alloys	* High material strength	* Corrosive	* Orthopedic implants, screws, pins, and plates
E.g.,: gold, platinum, titanium, steel, chromium, cobalt	* Easy to fabricate and sterilize	* Aseptic loosening	
		* Excessive elastic modulus	
Ceramics and carbon compounds	* High material strength	* Difficult to mold	* Bioactive orthopedic implants
E.g.,: calcium phosphate salts (HA), glass, oxides of aluminum and titanium	* Biocompatibility	* Excessive elastic modulus	* Dental implants
	* Corrosion resistance		* Artificial hearing aids
Polymers	* Biodegradable	* Leachable in body fluids	* Orthopedic and dental implants
	* Biocompatible	* Hard to sterilize	* Prostheses
	* Easily moldable and readily available		* Tissue engineering scaffolds
E.g.,: PMMA^*^, Polycaprolactone(PCL), PLA, polycarbonates, polyurethanes	* Suitable mechanical strength		* Drug delivery systems
Composites	* Excellent mechanical properties	* Expensive	* Porous orthopedic implants
E.g.,: Dental filling composites, carbon fiber reinforced methyl methacrylate bone cement + ultra-high molecular weight polyethylene	* Corrosive resistant	* Laborious manufacturing methods	* Dental fillings
			* Rubber catheters and gloves

* PMMA—poly (methyl methacrylate).

**Table 4:** Overview of the biomaterials used for FDM based 3D printing.

Materials	Fabrication Process	In Vivo/In Vitro Model	Key Findings	Ref.
Scaffolds for tissue engineering and regeneration				
PCL + Chitosan	Porous PCL scaffolds were 3D printed at 130 °C, print head speed of 1–3 mm/s and 1.5–3.0 bar pressure. Thermosensitive chitosan hydrogel was filled inside the pores of PCL scaffold.	Rabbit bone marrow mesenchymal stem cells (BMMSCs)	3D printed scaffolds showed greater cell retention and proliferation of BMMSCs. Stronger osteogenesis and higher bone matrix formation shows their applications in bone tissue engineering	[8]
PCL + β-TCP	PCL melted at 110 °C and β-TCP powder is added. Membranes were 3D printed at 110 °C and at 500 kPa.	Alveolar bone defects on beagles	The 3D printed PCL/β-TCP membranes showed enhanced bone regeneration capabilities than PCL or collagen membranes alone	[9]
PLA + biodegradable calcium phosphate glass	Printing pressure 40–80 psi, 3 mm/s motor speed, print head temperature 40 ± 5 °C, Cross-linked with 8% (*w/v*) NaOH in 70% ethanol.	Human monocytes	PLA based scaffolds increased the production of IL-6, IL-12/23 and IL-10	[19]
Drug Delivery				
PCL	Extruded PCL filaments with female sex hormones (E1, E2, E3 and progesterone) at 90 °C and 3D printed at 110 °C in the shape of commonly used implants including discs, pessaries, subdermal rods, intrauterine devices (IUDs) and surgical mesh.	Estrogen receptor luciferase reporter cells (T47D)	FDM can be used to fabricate patient specific personalized medicine for drug delivery. The 3D printed hormonal constructs showed biocompatibility and bioactive retention	[12]
PLA	PLA pellets coated with gentamicin and methotrexate were extruded as filaments at 170 °C and 3D printed as beads and catheters using Makerbot 3D printer (FDM based) at 220 °C	Osteosarcoma cells (for chemotherapeutics) and *E. coli* (for antibiotics)	3D printed PLA constructs successfully retained the bioactivity. Clear demarcating zones of inhibition was seen for gentamicin constructs and decrease in cell viability of osteosarcoma cells proved the cytostatic effect of methotrexate constructs.	[11]
Olea-gum-resins (benzoin, myrrha and olibanum) doped with metal oxide nanoparticles (TiO2, P25, Cu2O, and MoO3)	Natural gum resins added with 10% metal oxides were extruded as filaments at 70–85 °C and 3D printed into discs (10 mm × 5 mm) at 80 °C while maintaining the build platform temperature at 60 °C and at a print head speed of 10 mm/min.	*Staphylococcus aureus, Pseudomonas aeruginosa, Escherichia coli*, and *Candida albicans*.	Naturally occurring polymers can be successfully 3D printed. Discs with just the resins prevented only surface associated microbial growth. Additionally, metal oxide nanoparticles increased the bacteriostatic effects of the natural polymers	[13]
PVA	PVA filament was milled and powdered. Paracetamol and caffeine were added and extruded as filaments at 180 °C. These filaments were 3D printing into tablets and capsules at 200 °C with print head speed of 150 mm/s		Novel oral dosage forms were successfully fabricated. Capsules with alternating layers of caffeine and paracetamol were 3D printed.	[16]
Surgical guides and implants				
ABS	CAD models were developed using CT files of patient and 3D printed. FDM fabricated models were scanned again for comparison	Perioperative surgical simulation of conjoined twin separation surgery	The 3D printed models resembled the CT data of the patients and had an overall mean deviation of less than 2 mm.	[17]
TPU *	Pharmaceutical grade TPU powder was extruded into filaments and 3D printed into fistula stents, which were modelled from patient’s 3D reconstructed fistulography and CT scan images	A 45-year-old man was implanted with this tailor-made fistula implant	The 3D printed implant was effective in treating the enterocutaneous fistula	[18]

* TPU—thermoplastic urethane.

**Table 5:** Biomaterials used for extrusion based bioprinting.

Materials	Process	In Vivo/In Vitro Model	Key Findings	Ref.
Gelatin (partially crosslinked)	The partially polymerized gel in the print head was extruded at 30 °C through a 100 µm diameter nozzle on to a cooled platform (10 °C). These were later crosslinked with chemicals EDC/NHS * for thermal and mechanical stability. Sterilization was done by overnight incubation in 70% ethanol and one hour of UV exposure.	CD-1 strain (Harlan) female mice	3D printed implant restored ovarian function in the sterilized mice. Additionally, these mice successfully bore offspring.	[21]
Nano-fibrillated cellulose (NFC) + alginate	Using regenHU bioprinter, scaffolds (4.8 mm × 4.8 mm × 1 mm) were printed at printing pressure 40 kPa and 5 mm/s printing speed. Crosslinked using CaCl_2_ for 10 min, followed by rinsing with culture medium.	Human nasoseptal chondrocytes	Successfully 3D printed constructs resembling human organs (ear). The cytotoxicity and cell viability analysis proved the biocompatibility of this novel hydrogel (bioink) formulation.	[22]
NFC + alginate; NFC + HA	RegenHu bioprinter was used to 3D print the constructs of 7 mm × 7 mm × 1.2 mm dimensions with the two bioinks loaded with iPSCs. Printing speed was maintained at 10–20 mm/s at 20–30 kPa printing pressure. NFC-alginate constructs were crosslinked with CaCl_2_ for 5 min and NFC–HA constructs were crosslinked for 5 min using H_2_O_2_.	Human derived induced pluripotent stem cells (iPSCs)	The iPSCs in NFC-alginate constructs were pluripotent for at least 5 weeks, and then formed into hyaline like cartilage expressing type II collagen. NFC-hyaluronic acid constructs have shown lower proliferation rate.	[23]
Methacrylated hyaluronic acid (MeHA)	MeHA was dissolved in culture medium along with photoinitiator Irgacure 2959. Porous cubic scaffolds were bioprinted using Bioscaffolder dispensing system 3D bioprinter and scaffolds were UV crosslinked at 1800 mJ/cm^2^.	Mesenchymal stromal cells	Bioprinted scaffolds maintained good cell viability for more than 3 weeks. Increased concentrations of MeHA promoted osteogenic differentiation.	[31]
PVA and phytagel (1:1)	Printing was done at room temperature with a print speed of 5 mm/s and flow rate of 6 mL/h on to a cold build plate (−78.5 °C). The scaffolds were stored at −25 °C for 15 h. Constructs were later coated with collagen, poly-l-lysine or gelatin	Human dermal fibroblast cells	PVA/phytagel hydrogel was successfully 3D printed cryogenically and have mechanical properties similar to soft tissue. Additionally, coating with natural polymers (chitosan or gelatin) increased the cell attachment of the fibroblasts	[24]
Biphasic calcium phosphate (HA/β-TCP = 60:40) + HPMC + Polyethylenimine + ZrO_2_	Extruded at pressure of 600 kPa and at printing speed of 100 mm/min. Samples were sintered at 1100 °C	Tested on osteoblast like sarcoma cells for cytotoxicity and hMSCs for differentiation potential of the scaffolds	Improved mechanical properties of scaffolds at 10% (*w*/*w*) of ZrO_2_ was reported along with improved BMP-2 expression	[32]
Calcium sulfate hydrate + mesoporous bioglass + PCL	Extruded under pressure of 2.2–3.6 bar and speed of 4.5–8.2 mm/s	In vitro evaluation on hBMSc cells and in vivo evaluation on rat model	Addition of bioglass promoted bone formation significantly in the animal model	[33]
Calcium silicate + Magnesium + PVA	Extruded using a 450 µm nozzle and printed at speed of 6 mm/s. Scaffolds were sintered at 1150 °C	In vitro testing on MC3T3 cells an in vivo evaluation on rabbit skull defects	Mechanical strength was significantly improved along with degradation rate and new bone formation	[34]

* EDC/NHS—(1-ethyl-3-(3-dimethylaminopropyl) carbodiimide hydrochloride)/N-hydroxysuccinimide; HPMC—(hydroxypropyl methylcellulose); hMSCs—(human mesenchymal stem cells); hBMSc—(human bone marrow stromal cells).

**Table 6:** Biomaterials used for inkjet printing.

Materials	Process	In Vivo/In Vitro Model	Key Findings	Ref.
Powders: hydroxyapatite + β-TCP); Binding liquid: (0.6 wt % PVA + 0.25 wt % Tween 80) and (8.75 wt % phosphoric acid + 0.25 wt % Tween 80)	Microporous cylindrical scaffolds (3 mm × 10 mm) were 3D printed using ZPrinter 250 printer at 0.1 mm powder thickness and 0.3 L/m^3^ binder spray velocity. Scaffolds were set to dry at 50 °C for 2 h.	Rabbit bone marrow stromal cells (BMSCs)	Constructs printed with phosphoric acid showed better fabrication accuracy and mechanical properties than constructs printed with PVA. Both binding liquids showed good cellular affinity with BMSCs.	[35]
Substrate: paper and polyethylene terephthalate (PET); Binding liquid: concentrated solution of paracetamol, theophylline, and caffeine	Concentrated drug solutions were selectively placed on the substrates at 30 °C, and at 10 µm dropping distance using dimatix materials printer (DMP) 2800 inkjet printer.		Active pharmaceutical ingredients were successfully 3D printed using inkjet technology. The accurate deposition and crystallization of the drugs can be highly controlled. Precise and personalized dosing of the drug substances is possible with this technology.	[36]
Powders: β-TCP + hydroxyapatite + dextrin; Binding liquid: water + glycerol	Powder bed thickness was maintained 100 µm at 0.006 m/s print head speed. Constructs were gradually heated up to 350 °C and sintered at 1200 °C for 4 h. Fibrin and BMP-2 were coated. Osteoblasts were seeded on the scaffolds.	Male Lewis rats	3D printed constructs with BMP-2 and osteoblast cells showed enhanced ectopic bone formation.	[38]
Powder: α-TCP; Binding liquid: 8.75 wt % phosphoric acid + 0.25 wt % Tween 80	Powder layer thickness 89 µm and binder liquid to powder ratio 0.46. Vancomycin and rifampin were added to the powder bed. Polylactic-*co*-glucolic acid (PLGA) was coated in some groups.	Female BALB/cJ mice	Unlike PMMA, co-delivery of drugs vancomycin and rifampin was possible with 3D printed constructs. Thus, significantly improving implant-associated osteomyelitis. Additional PLGA coating further prolonged the antibiotic release.	[39]
Binding liquid: Soluplus (co-polymer of PVC-PVA-PEG); Substrate: stainless steel microneedles	Drugs curcumin, 5-fluorouracil, cis-platin were added to the polymer and jetted as fine droplets (300 pL) on the needles at 1–5 m/s. Multiple coatings were given to acquire desired drug concentration.	Dermatomed porcine skin	Inkjet printing technology was proved effective in coating metallic microneedles for transdermal drug delivery.	[37]
Binding liquid: miconazole; Substrate: Gantrez AN 169 BF (poly (methyl vinyl ether-co-maleic anhydride)) microneedles	Miconazole in dimethyl sulfoxide was sprayed at a rate of 10 pL/droplet of solution. Drop spacing of 30 µm and 32.0 V jet voltage was used.	*Candida albicans*	Antifungal agents were successfully incorporated using inkjet printing technology and clear zone of inhibition was demonstrated. Fabricated constructs can be effectively used for transdermal treatment of cutaneous fungal infections.	[40]
Binding liquid: 2-pyrolidinone; Substrate: calcium sulfate hemihydrate	89 µm layer height	Osteoblast like sarcoma cells	Binder solution toxicity was assayed by sintering specimens at temperature ranging from 300–1100 °C. High temperature sintered samples were compatible	[41]
Binding liquid: 8.75% phosphoric acid + 0.25% Tween80 + 1%–2% collagen; Substrate: hydroxyapatite and α-TCP	89 µm layer height and binding liquid to powder ratio was 0.46 was used	In vitro cytocompatibility was tested on C3H/10T1/2 cells and in vivo evaluation was done on critical size femoral defects on female BLAB/cJ	Macroporosity up to 0.5 mm was achieved. Incorporation of collagen favored better cellular response and improved mechanical properties.	[42]
Binding liquid: aqueous solution of 2-pyrrolidone (zb63); Substrate: calcium sulfate (plaster), vinyl polymer and carbohydrate	Pore sizes of 0.4, 0.6, and 0.8 mm were designed and printed at binder to powder ratio of 0.24 (shell) and 0.12 (core)	Effect of layer thickness and orientation of printing were evaluated by measuring physical and mechanical properties	Layer thickness of 0.1125 mm and printing along X direction resulted in specimens with best mechanical strength and dimensional accuracy	[43]
Binder liquid: mesoporous silica nanoparticles, polyethyleneimine, furosemide, and propylene glycol; Substrate: hydroxypropyl methyl cellulose (HPMC), and polyester transparency films	Print speed at 200 mm/s, resolution of 150 and 500 dpi, and wet thickness of 500 µm	Drug release from inks, rheological properties, dynamic viscosity and other important properties were evaluated	Successfully demonstrated the feasibility of printing drug loaded nano particle suspension for poorly water-soluble drugs	[44]

**Table 7:** Biomaterials used for polyjet printing.

Materials	Process	Test Model	Key Findings	Ref.
Elastic photopolymer (FullCure 930 TangoPlus) by Stratasys	3D printed live size aortic aneurysum phantom from patients CT files using a Stratasys Eden 260 polyjet printer. The phantom cost was $254.49 and took 13 hours to 3D print.	Mock surgical procedure was performed under live fluoroscope using the 3D printed phantom	Pre-surgical planning & simulation was possible with patient-specific abdominal aortic aneurysm phantom. Simulation was effective in planning surgical challenges & complications than standard procedures (2D image diagnostics).	[45]
Rigid acrylic resin (AR-M2) for Agilista-3200 3D printer, Japan	3D printed patient-specific intrahepatic vessel models	Preoperative planning in hepatocellular carcinoma resection procedure	The use of 3D printed intrahepatic vessel models from patient’s data (CT files) has greatly improved the surgical quality of the hepatocellular carcinoma procedure.	[46]
Photopolymer resin	3D printed customized surgical aids (cutting and repositioning guides) for genioplasty. CAD/CAM models were created from the patients CT images and patient specific surgical guides were fabricated using SLA based 3D printer (3D systems).	Genioplasty performed on 88 patients with dentofacial deformities	3D printed genioplasty templates provided greater accuracy in the surgical procedures than traditional intraoperative measurements.	[47]
Multiple photopolymer resins on Connex 3 polyjet	Printed at 16 µm layer height	3D printed anatomical phantoms of liver and microspheres from patient’s CT data	These phantoms offered a method to quantify radiation dose form Y-90 microspheres for treatment of liver cancer	[48]
Multiple photopolymer resins printed using Connex 350	Printed anatomical liver with different materials for vasculature and biliary structures	Used as preoperative surgical guidance model for 3 cases of liver transplant	6 patient specific liver models were 3D printed (3 living donor and 3 recipients). Significantly improved surgery and minimized intraoperative complications.	[49]
Multiple photopolymer resins printed using Objet 500 Connex	Printed anatomical model of head with different materials for skin, bone and tissues	Used these models as a training tool for neuro surgery	Significantly improved the training experience of surgeons by improving navigation and planning	[50]
Photopolymer RGD525 and Connex 500	Printed with polymers that are visible under MRI scanners	Spine model containing C6–C8 vertebrae including tumors in them.	Anatomically accurate phantoms that can be imaged under CT and MRI were developed. Improving preoperative planning for MR guided minimally invasive surgeries.	[51]
Multiple photopolymers and Objet 350 Connex	Materials with different rigidity were used to mimic native tissue’s mechanical properties.	Different models such as hollow aneurysm, craniocerebral aneurysm, and craniocerebral tumors	Aneurysm clippings and tumor resection planning were efficiently planned with these models	[52]
Multiple photopolymers and Objet studio	Materials with different flexibilities were used	50 patients were randomly chosen to explain medical procedure using 3D printed model	3D printed model of nasal sinus anatomy was used as educational tool to enable patients to make informed decision. Results suggest improved patient comfort levels and outcomes.	[53]
Projet 3512 HD	Rigid material was used to create molds for nephrology sectioning.	5 patient specific slicing guides were 3D printed for partial nephrectomy	Enabled accurate sectioning of tumors for colocalization analysis for radiomic and radiogenomic analyses	[54]

## References

[1] Belhabib S., Guessasma S. (2017). Compression performance of hollow structures: From topology optimisation to design 3D printing. Int. J. Mech. Sci..

[2] Guessasma S., Nouri H., Roger F. (2017). Microstructural and Mechanical Implications of Microscaled Assembly in Droplet-based Multi-Material Additive Manufacturing. Polymers.

[3] Ligon S.C., Liska R., Stampfl J., Gurr M., Mülhaupt R. (2017). Polymers for 3D Printing and Customized Additive Manufacturing. Chem. Rev..

[4] Liu T., Guessasma S., Zhu J., Zhang W., Nouri H., Belhabib S. (2018). Microstructural defects induced by stereolithography and related compressive behaviour of polymers. J. Mater. Process. Technol..

[5] Mandrycky C., Wang Z., Kim K., Kim D.H. (2016). 3D bioprinting for engineering complex tissues. Biotechnol. Adv..

[6] Rezwan K., Chen Q.Z., Blaker J.J., Boccaccini A.R. (2006). Biodegradable and bioactive porous polymer/inorganic composite scaffolds for bone tissue engineering. Biomaterials.

[7] Godbey W.T., Atala A. (2002). In vitro systems for tissue engineering. Ann. N. Y. Acad. Sci..

[8] Dong L., Wang S.J., Zhao X.R., Zhu Y.F., Yu J.K. (2017). 3D-printed poly (ϵ-caprolactone) scaffold integrated with cell-laden chitosan hydrogels for bone tissue engineering. Sci. Rep..

[9] Shim J.-H., Won J.-Y., Park J.-H., Bae J.-H., Ahn G., Kim C.-H., Lim D.-H., Cho D.-W., Yun W.-S., Bae E.-B. (2017). Effects of 3D-Printed Polycaprolactone/β-Tricalcium Phosphate Membranes on Guided Bone Regeneration. Int. J. Mol. Sci..

[10] Mills D., Tappa K., Jammalamadaka U., Weisman J., Woerner J. (2017). The Use of 3D Printing in the Fabrication of Nasal Stents. Inventions.

[11] Weisman J.A., Nicholson J.C., Tappa K., Jammalamadaka U., Wilson C.G., Mills D.K. (2015). Antibiotic and chemotherapeutic enhanced three-dimensional printer filaments and constructs for biomedical applications. Int. J. Nanomed..

[12] Tappa K., Jammalamadaka U., Ballard D.H., Bruno T., Israel M.R., Vemula H., Meacham J.M., Mills D.K., Woodard P.K., Weisman J.A. (2017). Medication eluting devices for the field of OBGYN (MEDOBGYN): 3D printed biodegradable hormone eluting constructs; a proof of concept study. PLoS ONE.

[13] Horst D.J., Tebcherani S.M., Kubaski E.T., De Almeida Vieira R. (2017). Bioactive Potential of 3D-Printed Oleo-Gum-Resin Disks: *B. papyrifera*; *C. myrrha*; and *S. benzoin* Loading Nanooxides—TiO_2_, P25, Cu_2_O; and MoO_3_. Bioinorg. Chem. Appl..

[14] Weisman J., Jammalamadaka U., Tappa K., Mills D. (2017). Doped Halloysite Nanotubes for Use in the 3D Printing of Medical Devices. Bioengineering.

[15] Goyanes A., Det-Amornrat U., Wang J., Basit A.W., Gaisford S. (2016). 3D scanning and 3D printing as innovative technologies for fabricating personalized topical drug delivery systems. J. Control. Release.

[16] Goyanes A., Wang J., Buanz A., Martínez-Pacheco R., Telford R., Gaisford S., Basit A.W. (2015). 3D Printing of Medicines: Engineering Novel Oral Devices with Unique Design and Drug Release Characteristics. Mol. Pharm..

[17] Shen S., Wang H., Xue Y., Yuan L., Zhou X., Zhao Z., Dong E., Liu B., Liu W., Cromeens B. (2017). Freeform fabrication of tissue-simulating phantom for potential use of surgical planning in conjoined twins separation surgery. Sci. Rep..

[18] Huang J.-J., Ren J.-A., Wang G.-F., Li Z.-A., Wu X.-W., Ren H.-J., Liu S. (2017). 3D-printed “fistula stent” designed for management of enterocutaneous fistula: An advanced strategy. World J. Gastroenterol..

[19] Almeida C.R., Serra T., Oliveira M.I., Planell J.A., Barbosa M.A., Navarro M. (2014). Impact of 3-D printed PLA- and chitosan-based scaffolds on human monocyte/macrophage responses: Unraveling the effect of 3-D structures on inflammation. Acta Biomater..

[20] Rhee S., Puetzer J.L., Mason B.N., Reinhart-King C.A., Bonassar L.J. (2016). 3D Bioprinting of Spatially Heterogeneous Collagen Constructs for Cartilage Tissue Engineering. ACS Biomater. Sci. Eng..

[21] Laronda M.M., Rutz A.L., Xiao S., Whelan K.A., Duncan F.E., Roth E.W., Woodruff T.K., Shah R.N. (2017). A bioprosthetic ovary created using 3D printed microporous scaffolds restores ovarian function in sterilized mice. Nat. Commun..

[22] Markstedt K., Mantas A., Tournier I., Martínez Ávila H., Hägg D., Gatenholm P. (2015). 3D Bioprinting Human Chondrocytes with Nanocellulose–Alginate Bioink for Cartilage Tissue Engineering Applications. Biomacromolecules.

[23] Nguyen D., Hägg D.A., Forsman A., Ekholm J., Nimkingratana P., Brantsing C., Kalogeropoulos T., Zaunz S., Concaro S., Brittberg M. (2017). Cartilage Tissue Engineering by the 3D Bioprinting of iPS Cells in a Nanocellulose/Alginate Bioink. Sci. Rep..

[24] Tan Z., Parisi C., Di Silvio L., Dini D., Forte A.E. (2017). Cryogenic 3D Printing of Super Soft Hydrogels. Sci. Rep..

[25] Lee J.-S., Hong J.M., Jung J.W., Shim J.-H., Oh J.-H., Cho D.-W. (2014). 3D printing of composite tissue with complex shape applied to ear regeneration. Biofabrication.

[26] Phillippi J.A., Miller E., Weiss L., Huard J., Waggoner A., Campbell P. (2008). Microenvironments Engineered by Inkjet Bioprinting Spatially Direct Adult Stem Cells Toward Muscle- and Bone-Like Subpopulations. Stem Cells.

[27] Duan B., Hockaday L.A., Kang K.H., Butcher J.T. (2013). 3D bioprinting of heterogeneous aortic valve conduits with alginate/gelatin hydrogels. J. Biomed. Mater. Res. A.

[28] Fedorovich N.E., Alblas J., de Wijn J.R., Hennink W.E., Verbout A.J., Dhert W.J.A. (2007). Hydrogels as Extracellular Matrices for Skeletal Tissue Engineering: State-of-the-Art and Novel Application in Organ Printing. Tissue Eng..

[29] Hsieh F.-Y., Lin H.-H., Hsu S. (2015). 3D bioprinting of neural stem cell-laden thermoresponsive biodegradable polyurethane hydrogel and potential in central nervous system repair. Biomaterials.

[30] Suntornnond R., An J., Chua C.K. (2017). Roles of support materials in 3D bioprinting. Int. J. Bioprint..

[31] Poldervaart M.T., Goversen B., de Ruijter M., Abbadessa A., Melchels F.P.W., Öner F.C., Dhert W.J.A., Vermonden T., Alblas J. (2017). 3D bioprinting of methacrylated hyaluronic acid (MeHA) hydrogel with intrinsic osteogenicity. PLoS ONE.

[32] Sa M.-W., Nguyen B.-N.B., Moriarty R.A., Kamalitdinov T., Fisher J.P., Kim J.Y. (2017). Fabrication and evaluation of 3D printed BCP scaffolds reinforced with ZrO_2_ for bone tissue applications. Biotechnol. Bioeng..

[33] Qi X., Pei P., Zhu M., Du X., Xin C., Zhao S., Li X., Zhu Y. (2017). Three dimensional printing of calcium sulfate and mesoporous bioactive glass scaffolds for improving bone regeneration in vitro and in vivo. Sci. Rep..

[34] Sun M., Liu A., Shao H., Yang X., Ma C., Yan S., Liu Y., He Y., Gou Z. (2016). Systematical Evaluation of Mechanically Strong 3D Printed Diluted magnesium Doping Wollastonite Scaffolds on Osteogenic Capacity in Rabbit Calvarial Defects. Sci. Rep..

[35] Wang Y., Wang K., Li X., Wei Q., Chai W., Wang S., Che Y., Lu T., Zhang B. (2017). 3D fabrication and characterization of phosphoric acid scaffold with a HA/β-TCP weight ratio of 60:40 for bone tissue engineering applications. PLoS ONE.

[36] Sandler N., Määttänen A., Ihalainen P., Kronberg L., Meierjohann A., Viitala T., Peltonen J. (2011). Inkjet printing of drug substances and use of porous substrates-towards individualized dosing. J. Pharm. Sci..

[37] Uddin M.J., Scoutaris N., Klepetsanis P., Chowdhry B., Prausnitz M.R., Douroumis D. (2015). Inkjet printing of transdermal microneedles for the delivery of anticancer agents. Int. J. Pharm..

[38] Strobel L.A., Rath S.N., Maier A.K., Beier J.P., Arkudas A., Greil P., Horch R.E., Kneser U. (2014). Induction of bone formation in biphasic calcium phosphate scaffolds by bone morphogenetic protein-2 and primary osteoblasts. J. Tissue Eng. Regen. Med..

[39] Inzana J.A., Trombetta R.P., Schwarz E.M., Kates S.L., Awad H.A. (2015). 3D printed bioceramics for dual antibiotic delivery to treat implant-associated bone infection. Eur. Cells Mater..

[40] Boehm R.D., Miller P.R., Daniels J., Stafslien S., Narayan R.J. (2014). Inkjet printing for pharmaceutical applications. Mater. Today.

[41] Asadi-Eydivand M., Solati-Hashjin M., Shafiei S.S., Mohammadi S., Hafezi M., Osman N.A.A. (2016). Structure; properties; and in vitro behavior of heat-treated calcium sulfate scaffolds fabricated by 3D printing. PLoS ONE.

[42] Inzana J.A., Olvera D., Fuller S.M., Kelly J.P., Graeve O.A., Schwarz E.M., Kates S.L., Awad H.A. (2014). 3D printing of composite calcium phosphate and collagen scaffolds for bone regeneration. Biomaterials.

[43] Farzadi A., Solati-Hashjin M., Asadi-Eydivand M., Osman N.A.A. (2014). Effect of layer thickness and printing orientation on mechanical properties and dimensional accuracy of 3D printed porous samples for bone tissue engineering. PLoS ONE.

[44] Wickström H., Hilgert E., Nyman J., Desai D., Şen Karaman D., de Beer T., Sandler N., Rosenholm J. (2017). Inkjet Printing of Drug-Loaded Mesoporous Silica Nanoparticles—A Platform for Drug Development. Molecules.

[45] Meess K.M., Izzo R.L., Dryjski M.L., Curl R.E., Harris L.M., Springer M., Siddiqui A.H., Rudin S., Ionita C.N. (2017). 3D Printed Abdominal Aortic Aneurysm Phantom for Image Guided Surgical Planning with a Patient Specific Fenestrated Endovascular Graft System. Proc. SPIE Int. Soc. Opt. Eng..

[46] Kuroda S., Kobayashi T., Ohdan H. (2017). 3D printing model of the intrahepatic vessels for navigation during anatomical resection of hepatocellular carcinoma. Int. J. Surg. Case Rep..

[47] Li B., Wei H., Zeng F., Li J., Xia J.J., Wang X. (2017). Application of A Novel Three-dimensional Printing Genioplasty Template System and Its Clinical Validation: A Control Study. Sci. Rep..

[48] Gear J.I., Cummings C., Craig A.J., Divoli A., Long C.D.C., Tapner M., Flux G.D. (2016). Abdo-Man: A 3D-printed anthropomorphic phantom for validating quantitative SIRT. EJNMMI Phys..

[49] Zein N.N., Hanouneh I.A., Bishop P.D., Samaan M., Eghtesad B., Quintini C., Miller C., Yerian L., Klatte R. (2013). Three-dimensional print of a liver for preoperative planning in living donor liver transplantation. Liver Transplant..

[50] Waran V., Narayanan V., Karuppiah R., Owen S.L.F., Aziz T. (2014). Utility of multimaterial 3D printers in creating models with pathological entities to enhance the training experience of neurosurgeons. J. Neurosurg..

[51] Mitsouras D., Lee T.C., Liacouras P., Ionita C.N., Pietilla T., Maier S.E., Mulkern R.V. (2017). Three-dimensional printing of MRI-visible phantoms and MR image-guided therapy simulation. Magn. Reson. Med..

[52] Lan Q., Chen A., Zhang T., Li G., Zhu Q., Fan X., Ma C., Xu T. (2016). Development of Three-Dimensional Printed Craniocerebral Models for Simulated Neurosurgery. World Neurosurg..

[53] Sander I., Liepert T., Doney E., Leevy W., Liepert D. (2017). Patient Education for Endoscopic Sinus Surgery: Preliminary Experience Using 3D-Printed Clinical Imaging Data. J. Funct. Biomater..

[54] Dwivedi D.K., Chatzinoff Y., Zhang Y., Yuan Q., Fulkerson M., Chopra R., Brugarolas J., Cadeddu J.A., Kapur P., Pedrosa I. (2017). Development of a Patient-specific Tumor Mold Using Magnetic Resonance Imaging and 3-Dimensional Printing Technology for Targeted Tissue Procurement and Radiomics Analysis of Renal Masses. Urology.

[55] Miller J.S., Stevens K.R., Yang M.T., Baker B.M., Nguyen D.-H.T., Cohen D.M., Toro E., Chen A.A., Galie P.A., Yu X. (2012). Rapid casting of patterned vascular networks for perfusable engineered three-dimensional tissues. Nat. Mater..

[56] Kolesky D.B., Truby R.L., Gladman A.S., Busbee T.A., Homan K.A., Lewis J.A. (2014). 3D bioprinting of vascularized; heterogeneous cell-laden tissue constructs. Adv. Mater..

[57] Hinton T.J., Jallerat Q., Palchesko R.N., Park J.H., Grodzicki M.S., Shue H.-J., Ramadan M.H., Hudson A.R., Feinberg A.W. (2015). Three-dimensional printing of complex biological structures by freeform reversible embedding of suspended hydrogels. Sci. Adv..

[58] Hassana B.O., Guessasma S., Belhabib S., Nouri H. (2016). Explaining the Difference between Real Part and Virtual Design of 3D Printed Porous Polymer at the Microstructural Level. Macromol. Mater. Eng..

[59] Ventola C.L. (2014). Medical Applications for 3D Printing: Current and Projected Uses. Pharm. Ther..

